# Multimodal data for behavioural authentication in Internet of Things environments

**DOI:** 10.1016/j.dib.2024.110697

**Published:** 2024-07-01

**Authors:** Andraž Krašovec, Gianmarco Baldini, Veljko Pejović

**Affiliations:** a University of Ljubljana, Faculty of Computer and Information Science, Večna pot 113, 1000 Ljubljana, Slovenia; b European Commission, Joint Research Centre (JRC), Via Enrico Fermi 2749, 21027 Ispra (VA), Italy; c Department of Computer Systems, Institute “Jožef Stefan”, Jamova cesta 39, 1000 Ljubljana, Slovenia

**Keywords:** Ubiquitous sensing, User authentication, Inertial measurement unit, Wireless ranging, Electroencephalogram, Cognitive load, Multi-modal sensing

## Abstract

Identifying humans based on their behavioural patterns represents an attractive basis for access control as such patterns appear naturally, do not require a focused effort from the user side, and do not impose the additional burden of memorising passwords. One means of capturing behavioural patterns is through passive sensors laid out in a target environment. Thanks to the proliferation of the Internet of Things (IoT), sensing devices are already embedded in our everyday surroundings and represent a rich source of multimodal data. Nevertheless, collecting such data for authentication research purposes is challenging, as it entails management and synchronisation of a range of sensing devices, design of diverse tasks that would evoke different behaviour patterns, storage and pre-processing of data arriving from multiple sources, and the execution of long-lasting user activities. Consequently, to the best of our knowledge, no publicly available datasets suitable for behaviour-based authentication research exist. In this brief article, we describe the first multimodal dataset for behavioural authentication research collected in a sensor-enabled IoT setting. The dataset comprises of high-frequency accelerometer, gyroscope, and force sensor data collected from an office-like environment. In addition, the dataset contains 3D point clouds collected with wireless radar and electroencephalogram (EEG) readings from a wireless EEG cap worn by the study participants. Within the environment, 54 volunteers conducted 6 different tasks that were constructed to elicit different behaviours and different cognitive load levels, resulting in a total of 16 h of multimodal data. The richness of the dataset comprising 5 different sensing modalities, a variability of tasks including keyboard typing, hand gesturing, walking, and other activities, opens a range of opportunities for research in behaviour-based authentication, but also the understanding of the role of different tasks and cognitive load levels on human behaviour.

Specifications Table

**Table:** 

Subject	Cryptography and Cybersecurity, Human-Computer Interaction
Specific subject area	Internet of Things sensors, behavioural patterns, cognitive load measurements
Data format	Raw data
Type of data	Table
Data collection	The following sensors were installed in an office-like environment: accelerometers and gyroscopes attached to a keyboard and a mouse, force sensors were placed under a plate, surrounding the working area, and a wireless radar placed under a computer monitor. In addition, a portable electroencephalogram cap was worn by study participants. In such an environment, 54 adult volunteers, one at a time, conducted 6 different tasks, where each user performed two seances of each of the tasks. Furthermore, each task was delivered in three flavours, where different flavours were designed to induce a different level of cognitive load.
Data source location	The data was collected at the European Commission's Joint Research Centre premises in Ispra, Italy.
Data accessibility	Repository name: Multimodal Environmental Data for Behavioural Authentication in Internet of Things Data identification number: 7be86ffd-1bac-4d3e-82fc-02b3ea40ab49 Direct URL to data: https://data.jrc.ec.europa.eu/dataset/7be86ffd-1bac-4d3e-82fc-02b3ea40ab49

## 1 Value of the Data

• The collected dataset will serve as a testing ground for future development of behavioural biometrics-based authentication algorithms that are based on passively collected sensor data from the environment.
• The multimodality of the collected data greatly exceeds that of the existing related datasets [[1], [2], [3]]. This will allow researchers to identify the most promising modalities whose future exploration would lead to the most reliable behavioural biometrics-based authentication.
• Our experimental protocol subjected participants to different levels of cognitive load while performing different tasks. The collected sensor data reflects behavioural change induced by varying cognitive load. Consequently, the data opens possibilities for further research on the impact of cognitive load on behaviour and, especially, the effect on behavioural properties relevant to sensor-based authentication.
• The data includes signals recorded using a wireless electroencephalogram (EEG). This is, to the best of our knowledge, the first open dataset containing EEG signals collected while participants were moving unrestrictedly in an office-like environment. As such, it opens opportunities for further development of data processing of EEG signals collected in unconstrained environments.
• The dataset also presents a valuable asset for wireless radar research, as for the first time radar data was collected as users were conducting a variety of office tasks under different cognitive strains. This will allow researchers to examine to what extent body movement modulated by tasks and cognitive load can be reflected in radar data.

## 2 Background

Authentication through one-off methods, such as typed passwords or even conventional biometrics, is increasingly becoming insufficient in dynamic environments with a multitude of interspersed users, such as hospitals, hotel reception desks, and factory floors, as the burden of frequent re-authentication may interfere with time-critical work in such environments [4]. While alternative biometric-based authentication exists, it harnesses sensitive information, including fingerprints, facial imaging, and voice recordings. Recently, behavioural biometrics emerged as a means of authenticating users from their inherent behavioural patterns [5,6]. These patterns can be reflected in non-sensitive data captured by sensors that are, thanks to the proliferation of the Internet of Things, already present in the environment. The non-sensitive aspect is reflected in the inherent resilience of such sensor data to privacy-related concerns, contrasting to the physical biometrics, such as fingerprints and facial recognition. Developing sophisticated machine learning algorithms that would enable user identification from such data is conditioned on the existence and availability of a dataset that includes multimodal data reflecting individuals’ behaviour. Furthermore, previous research has observed variability within a single user's behaviour [8], thus it is essential that future datasets also capture the properties (i.e. the difficulty) of a task that a person executes and include in-situ measurements of users’ internal state, for instance, their cognitive load.

## 3 Data Description

We publish the dataset in a collection of comma separated value (CSV) files in a structured folder directory. We define the following structure to separate the files:

<user_id>/<seance_id>/<experimental_interface>/ <experimental_task>/<difficulty>/

- **<user_id>** An incremented, zero-padded integer value starting from 1 that corresponds to a single participant of the experiments.
- **<seance_id>** Zero-padded integer value that represents an id of a single seance of a user. The numbers correspond to the actual seance id values in the database, therefore, the numbers do not start from one and are not always consecutive.
- **<experimental_interface>** Either *comp* or *tablet.* This corresponds to the device that the user interacts with during each performed experimental task. c*omp* stands for a desktop computer, navigation with keyboard and mouse, while *tablet* is a tablet computer, where the user relies on their fingers to complete the given tasks.
- **<experimental_task>***A* name of a specific experimental task that the participant performed.
- **<difficulty>** Designed difficulty level of a given experimental task. Either *lo* (low), *md* (medium), or *hg* (high).

Each leaf of this directory structure includes several files that contain raw sensor records of different sensing modalities as well as belonging responses to the NASA TLX questionnaire. The files are:

- **eeg_records.csv** Sensor records captured by the EEG helmet. It includes three columns; *sensor id* denotes to which channel of the helmet the record belongs to, *value* presents the actual value that was recorded by the sensor, and *time* holds the value of the timestamp of every record.
- **iot_records.csv** Sensor records captured by the inertial measurement unit (IMU) and force sensors. It includes the same columns as the file containing EEG records.
- **nasa_tlx.csv** Responses of the six dimensions of the NASA TLX questionnaire [7]. The file only includes a single row of data – responses given for each specific case of the experimental tasks.
- **radar_records.csv** 3d point clouds captured by the short-millimetre wave radar. Similarly to other record files, there are three columns present; *sensor id* can be ignored here, as there is only one radar gathering the point clouds. *radar pointcloud* is a string representation of a JSON-encoded array of points that belong to a point cloud. Each point consists of three values – x, y, and z coordinate of the point. Lastly, *time* again holds the timestamp value of each record. A JSON parsing function should be used to obtain the original array, e.g. the *json.loads* function in the Python programming language.

The only exception to the filing convention is the *Lock (PU)* experimental task, where there are no specific difficulties or sensor data involved. Instead, a single CSV file (*gestures.csv*) is present. This file holds gesture data of the unlock attempts in each seance. It consists of a single column that includes a JSON-encoded array of points. Each point holds consecutive x and y coordinates of the gesture that the user inputs. These points can be retrieved in the same way as the 3D point clouds of radar data.

## 4 Experimental Design, Materials and Methods

We build the experimental testbed based on our previous experiences in the field of sensor-based user authentication [8]. The main components of the testbed include the experimental tasks and the procedure that the volunteers have to complete, and the sensing infrastructure consisting of multiple sensor modalities and a data collection management backend.

### 4.1 Tasks

The volunteers have to complete six experimental tasks, each with three different difficulty levels, on two different devices (a PC and a tablet). We take three of the tasks from the elementary cognitive tasks (ECTs) suite [9] and develop the other three originally for this data collection campaign. The tasks include:

- **Writing (W) task**, where the user must retype a body of text consisting of around 500 characters displayed on the screen. The difficulty of the task Is modulated by selecting texts of different languages, based on the familiarity of an individual with a selected language: mother tongue, English, or Hungarian, where none of our volunteers is a native English speaker and none of the volunteers is familiar with Hungarian. The task is performed on the PC, and the time limit is set to one minute. The volunteer's goal is to retype as many words as possible within the given time limit.
- **Treasure hunt (TH) task**, where a participant must leave their working area, walk to a secondary screen, and memorise a number written on the screen. There is a ten-second time limit after which the number disappears. Next, the participant must return to their primary device and after thirty seconds they type the memorised number in the interface. The difficulty is modulated by the number of digits present in the number. The task is performed on the PC only, where the tablet serves as a secondary screen that is positioned approximately five meters away from the PC. The goal of the user is to memorise as many digits as possible in the correct order.
- **Pattern unlock (PU) task**, where the user must draw a predetermined nine-point pattern on a tablet to unlock it. This procedure is repeated ten times and is the only task that does not include different difficulty levels of cognitive load.
- **Hidden patterns (HP) task** is one of the ECTs where the user must identify a given pattern in one of the three images available. The difficulty is modulated by the complexity of the images. The task is performed on both the PC and the tablet and the time limit is set to thirty seconds. The goal of the volunteer is to correctly identify as many images as possible.
- **Number comparison (NC) task** is one of the ECTs where the user must compare pairs of numbers shown on a screen and mark a pair if the numbers in a pair are not equal. The difficulty is modulated by the number of digits in the number pairs. The task is performed on both the PC and the tablet, and the time limit is set to thirty seconds. The goal is to identify as many pairs of unequal numbers as possible.
- **Pursuit (P) task** is one of the ECTs where the user is presented with ten lines, entangled between themselves, each connected to a number on the left side and an empty box on the right side. The user's goal is to copy a number shown next to the left end of an entangled line to a corresponding box present at the right end. The difficulty is modulated by the level of the entanglement between lines. The task is only performed on the tablet and the time limit is set to one minute. The goal of the user is to correctly connect as many boxes as possible.

**Fig. 1:**
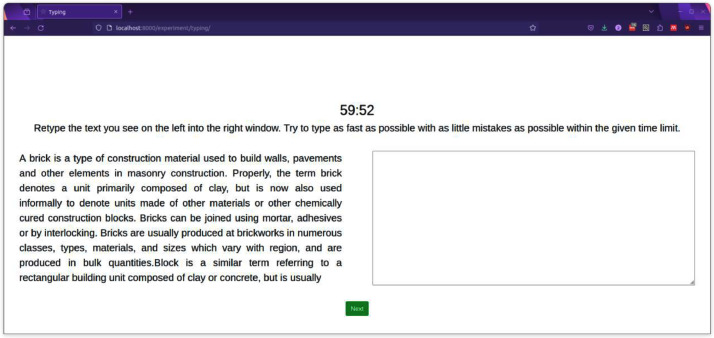
An example of the user interface during the writing task. The timer on top counts down to zero to push volunteers to apply during the experiments. All experiments run in a browser and are not dependent on a specific platform.

To motivate the volunteers to put effort into completing the tasks, we limit the time for completing each task. Furthermore, after they are done with the experiments, we display a leaderboard of previous attempts of other volunteers in an anonymised form and provide volunteers with feedback on how well they performed. A sample of the experimental interface is shown in Fig. 1.

### 4.2 Procedure

Each volunteer performed the experiments separately in sessions lasting at most 30 min. After welcoming a volunteer, we provide some basic information about the challenges they will have to complete and warn them about the dangers of EEG devices for people with active medical implant devices. Next, we present them with the Informed Consent (included in the submission), which they must read and sign. Additionally, we present them with the Privacy Policy of our data collection. Afterwards, we install the EEG helmet on the participant's head and perform an impedance test to confirm that the device has been installed correctly. To minimise the discrepancies in user performance between runs, each volunteer first performs a test run where they have a chance to experience each of the experimental tasks with instructions and explanations provided, after which the actual data collection begins. First, the user performs the tasks on the PC in the following order: W, HP, NC, TH. Then, the participant moves on to the tablet to conduct PU, P, HP, and NC. Except the PU task, each task is repeated three times, once for each difficulty level (easy, medium, and difficult). The order of difficulty levels is selected at random. To collect additional feedback on the level of cognitive load, the volunteers must fill in a NASA-TLX questionnaire after each difficulty level of each task performed. With everything complete, we reset the experiment, and, after a brief rest, the volunteers perform a second run of the tasks in the same order. Throughout the experiment, a member of our team was monitoring the sensor data collection through backend-based visualisations to ensure that no data loss or other unintended anomalies occurred.

### 4.3 Backend

The backend that ingests all sensor data is written in Python utilising the Django framework. Apart from the radar and EEG devices, all data is streamed via the MQTT communication protocol and inserted into the database in ten-second intervals. Separately, the radar data is captured via a USB connection to the data collection server while the EEG records are stored on the wireless EEG device and downloaded after the experiment. Both the radar and the EEG receive a time reference point at the start of each task, ensuring that data from all sensors is properly aligned in time. We implement custom scripts that ingest and align the data of these two devices after the experiments are completed.

### 4.4 Sensors

**Fig. 2:**
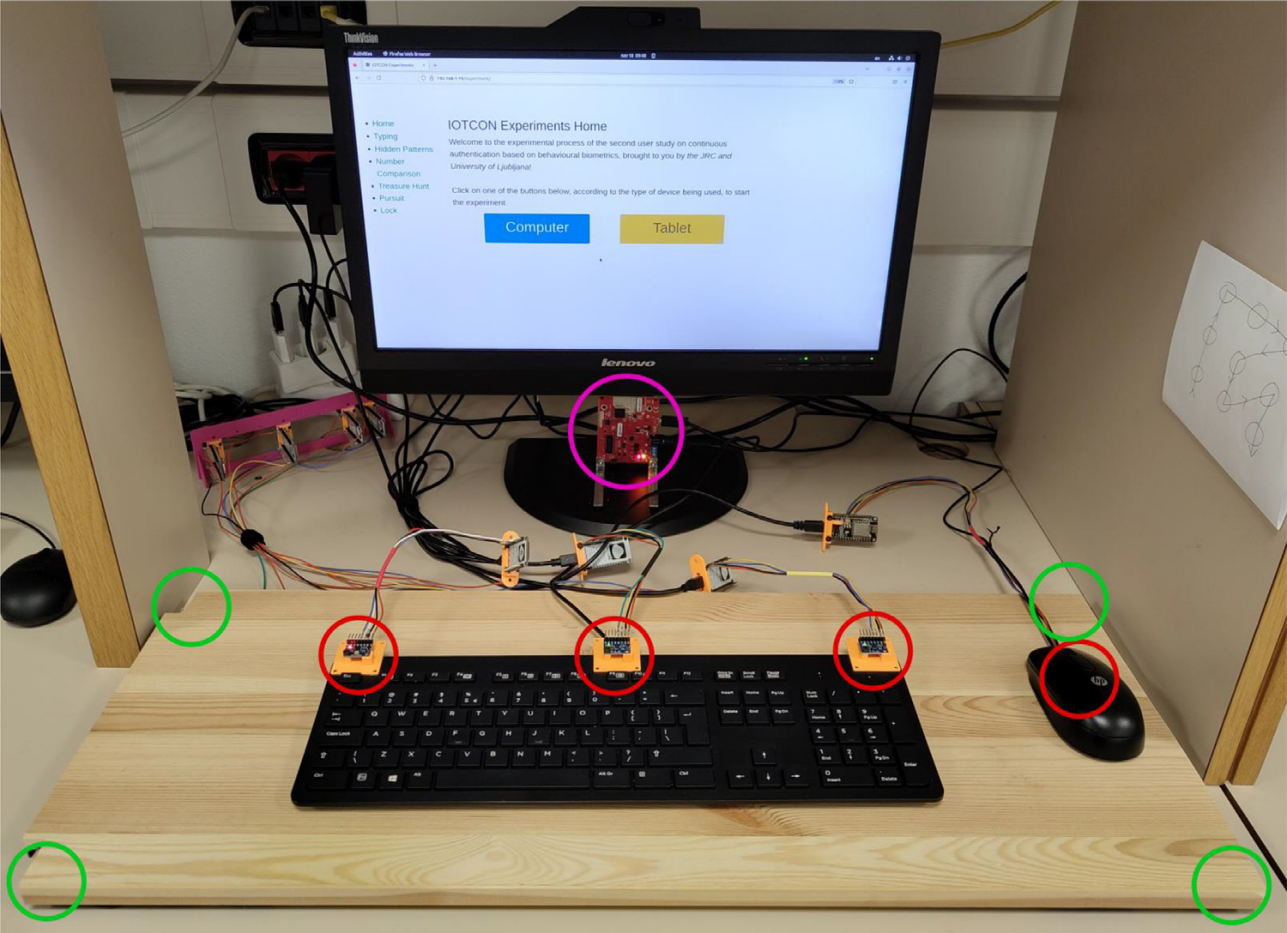
Experimental setup with the four IMU devices on the keyboard and in the mouse (red circles), force sensing resistors under the pressure plate (green circles) and the short millimetre wave radar positioned beneath the monitor (pink circle). (For interpretation of the references to color in this figure legend, the reader is referred to the web version of this article.)

**Fig. 3:**
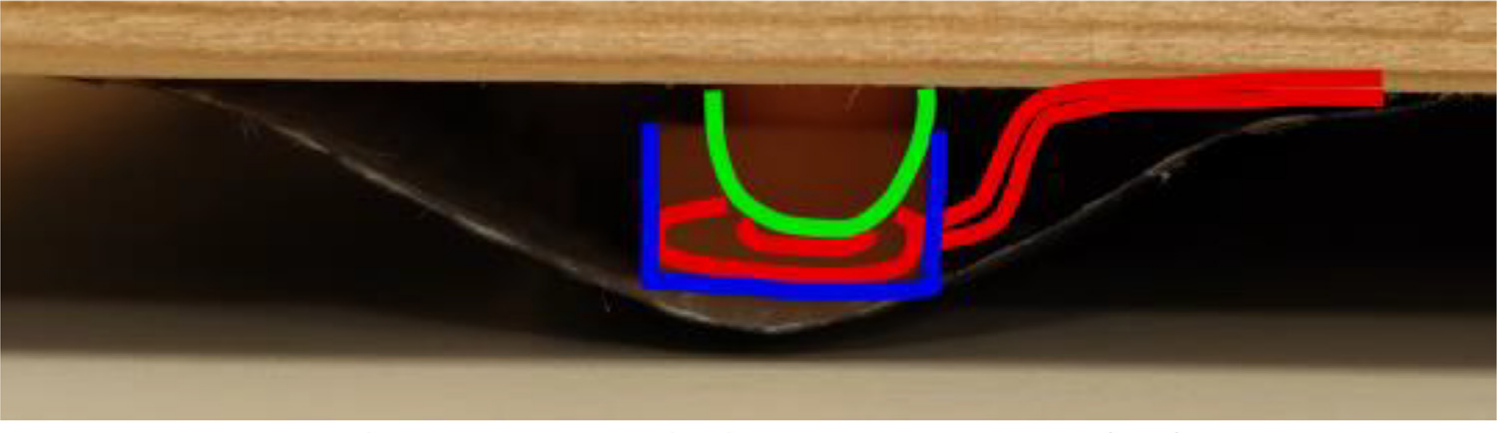
A closeup of the placement of a force-sensing resistor (red) with the pressure-inducing pin (green) and an enclosure (blue) that ensures that the force is applied in a repeatable fashion. (For interpretation of the references to color in this figure legend, the reader is referred to the web version of this article.)

**Fig. 4:**
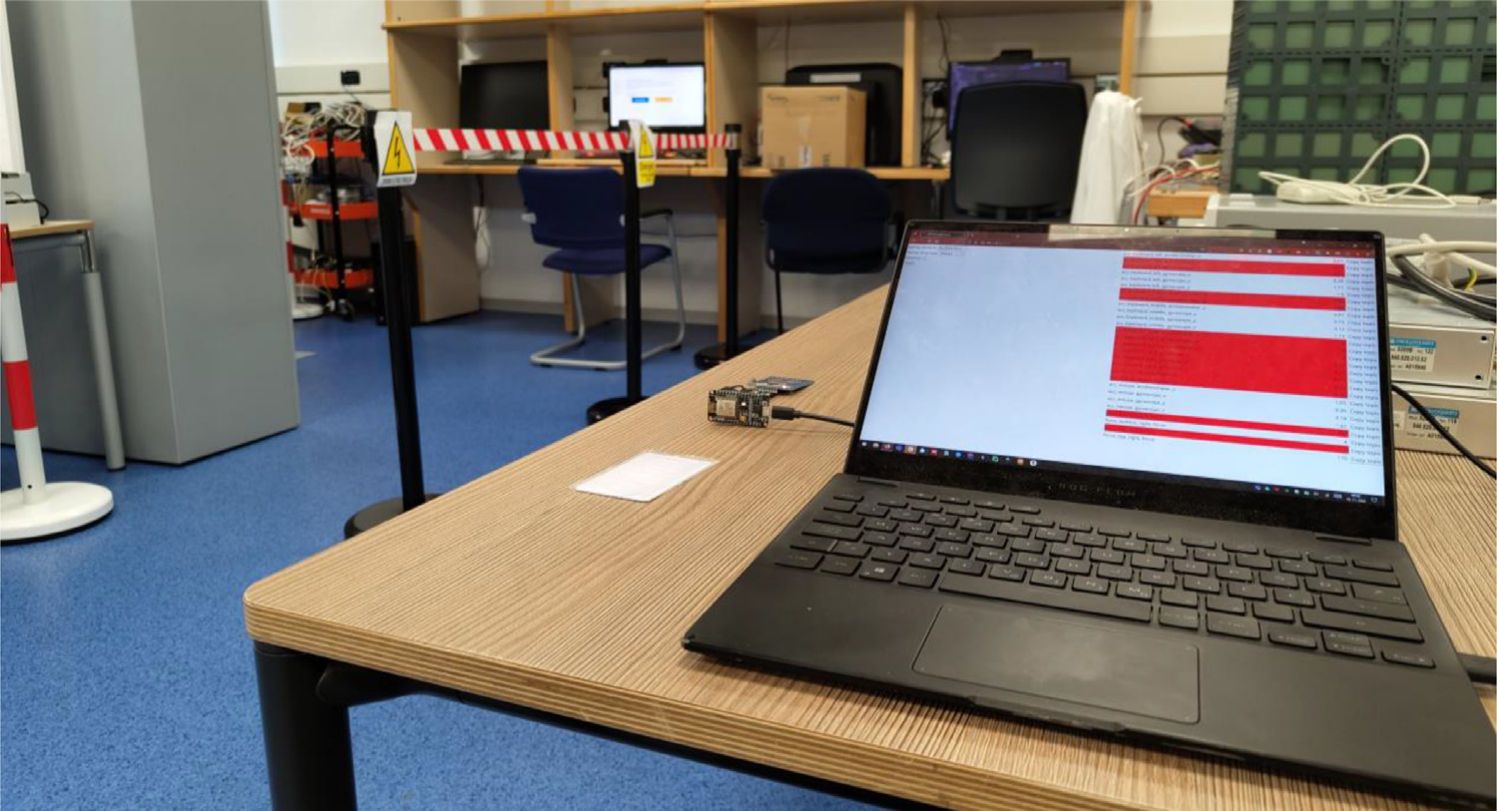
An image of the experimental testbed from the perspective of the operator. The laptop on the right is running the real-time monitoring software which helps the operator to immediately spot any potential issues. Each experimental run starts here with sensor initialisation. In the background the volunteer-operated PC is shown.

**Fig. 5:**
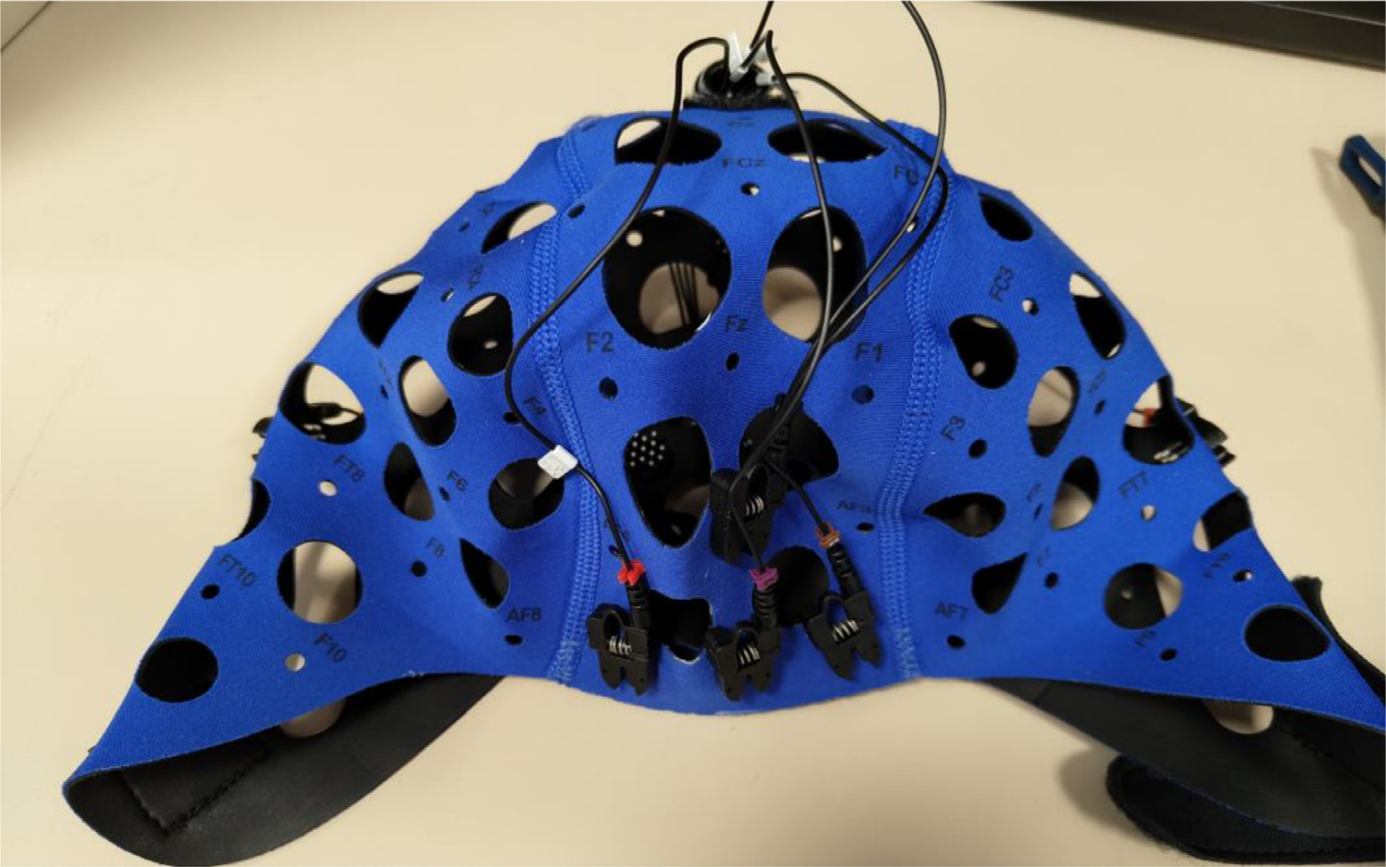
A front view of an electroencephalogram helmet with the four electrodes (three sensing channels and the ground) placed on the forehead of the volunteer.

**Fig. 6:**
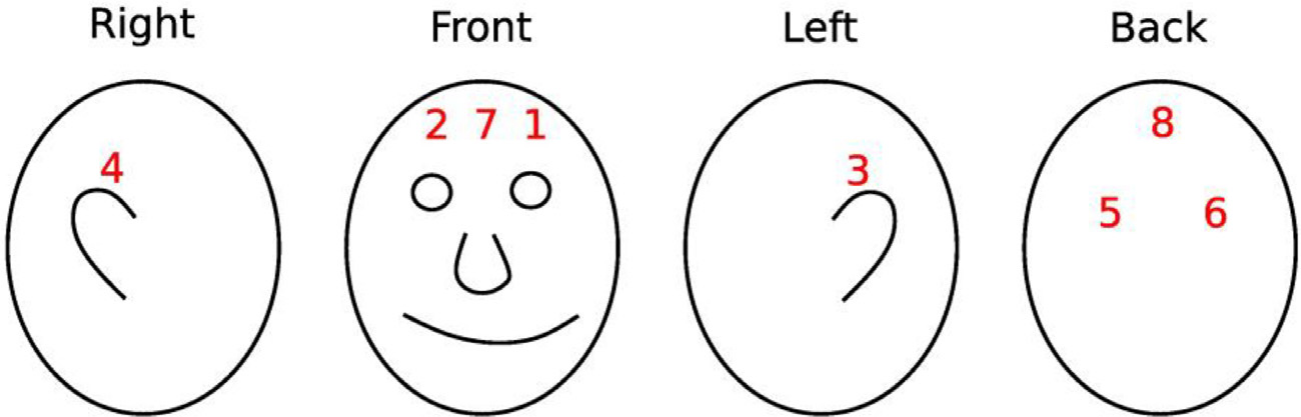
Positions of EEG electrodes on the user's head.

We utilise four different sensor modalities and ten sensor devices that together collect 39 sensor signals. Key specifications of the collected data are presented in Table 1, the sensor setup is displayed in Fig. 2 and an overview of the testbed in Fig. 4. Of the four inertial measurement units (MPU 4050) three are placed on the top part of the keyboard (left, middle, and right), while the fourth is integrated into the mouse that the users are interacting with. Each of these gathers acceleration and gyration in three axes. Underneath the working area of the user, which includes the keyboard and the mouse, we place a pressure-sensitive plate that has a force-sensitive resistor (df9–40) in each corner. Details on placement are displayed in Fig. 3. Each sensor measures the force exerted at a specific corner of the plate. A short millimetre-wave radar is placed below the monitor of the experimental PC, facing towards the user. It collects a 3D point cloud of the user while they move around the environment. Lastly, an eight-channel EEG helmet (Mentalab Explore^1^) that is installed on the volunteer's head before conducting the experiments measures the user's brain activity on the user's scalp. The helmet is shown in Fig. 5 and the positions of electrodes are displayed in Fig. 6.

**Table 1:** Specifications of the data captured by the sensors in the experimental testbed. The variable sampling frequency of the IWR1443 radar is a consequence of the required processing time and is dependent on the number of points in the cloud (more points, lower sampling rate).

Sensor	Number	Channels	Data Type	Data Range	Sampling Frequency
MPU 4050	4	Acceleration in x, y, z Gyration in x, y, z	Float	−5 – 5 (acceleration) −360 – 360(gyration)	200 Hz
DF9–40	4	Exerted force	Float	0 – 1024	200 Hz
TI IWR1443	1	3D point cloud	Array of coordinates	NA	1 – 200 Hz
Mentalab Explore	1	8-channel EEG	Float	−400,000 – 400,000	250 Hz

## Limitations

None.

## Ethics Statement

Relevant informed consent was obtained from the human subjects participating in the experiments. We append a copy of the consent form to this submission. The work was approved by the internal Ethical Committee of the Joint Research Centre, European Commission, ethics evaluation document number IMS-JRC-M1.2-TMP-0004.

## CRediT authorship contribution statement

**Andraž Krašovec:** Conceptualization, Methodology, Software, Validation, Investigation, Formal analysis, Data curation, Writing – original draft, Writing – review & editing, Visualization. **Gianmarco Baldini:** Conceptualization, Methodology, Writing – review & editing, Resources, Project administration. **Veljko Pejović:** Conceptualization, Methodology, Writing – original draft, Writing – review & editing, Resources, Project administration.

## Data Availability

Multimodal Environmental Data for Behavioural Authentication in Internet of Things (Original data) (Joint Research Centre Data Catalogue)

## Footnotes

1 https://www.mentalab.com/.
